# Gamma Irradiation and Ag and ZnO Nanoparticles Combined Treatment of Cotton Textile Materials

**DOI:** 10.3390/ma15082734

**Published:** 2022-04-08

**Authors:** Ovidiu-Alexandru Capraru, Bogdan Lungu, Marian Virgolici, Mihai Constantin, Mihalis Cutrubinis, Laura Chirila, Ludmila Otilia Cinteza, Ioana Stanculescu

**Affiliations:** 1 Horia Hulubei National Institute for Physics and Nuclear Engineering, IRASM Department, 30 Reactorului Str., 077125 Magurele, Romania; ovidiu.capraru@nipne.ro (O.-A.C.); ion.lungu@nipne.ro (B.L.); mvirgolici@nipne.ro (M.V.); mconstantin@nipne.ro (M.C.); mcutrubinis@nipne.ro (M.C.); 2 National Research & Development Institute for Textile and Leather, 16 Lucretiu Patrascanu Str., 030508 Bucharest, Romania; chirila_laura@yahoo.com; 3 Department of Physical Chemistry, University of Bucharest, 4-12 Regina Elisabeta Bld, 030018 Bucharest, Romania; ocinteza@gmail.com

**Keywords:** gamma irradiation, cotton, ATR, TG/DSC, nanocomposites

## Abstract

In this work, cotton textile materials were impregnated by immersion with three different nanocomposites: Ag/chitosan, Ag/polyvinylpyrrolidone, and ZnO/polyvinylpyrrolidone and irradiated with a ^60^Co gamma source. After the nanoparticles impregnation, the cotton materials were irradiated in a dry and wet state at 5 and 20 kGy radiation doses. The following methods were used for the characterization of the obtained cotton materials to reveal the modification of the textile materials: Fourier transform infrared-attenuated total reflection spectroscopy (FTIR-ATR) and thermogravimetry (TG). The obtained materials have good antibacterial properties. The microbiological tests have shown the best material results for the gamma irradition and Ag nanoparticles combined treatment. The objective was to create a more environmentally friendly approach for textile functionalization by eliminating toxic chemicals-based technology and replacing it with the eco-friendlier gamma technology.

## 1. Introduction

Textiles are some of the most used materials in the world with a wide range of applications. Their diversified use in domains such as medicine [1], clothing [2], and electronics [3] have transformed the textile industry. Classic methods of production are insufficient to satisfy the demands, so new techniques and apparatuses are needed to comply to the increased needs. The interest in developing new textiles with “smart properties” is very high [4,5] due to, among others, the evolution of antibiotic-resistant bacteria. Thus, cotton gauzes, medical gowns, and hospital bed sheets are just some examples of cotton textiles that could favor bacterial growth due to their moisture absorbing property.

The ionic state of Ag^+^ has an antimicrobial effect and is used as a common microbial inhibitor in various commercial and medical products [6]. Furthermore, silver nanoparticles showed antibacterial activity against *E. coli*, *P. aeruginosa*, and methicillin-resistant *S. aureus* [7]. A synergistic antibacterial effect was observed even when silver nanoparticles were combined with other natural and synthetic compounds [8]. The method of obtaining stable dispersions of Ag and ZnO nanoparticles relied on using PVP (polyvinylpyrrolidone) and chitosan as dispersing matrices. Gamma irradiation offers an alternative to the classical approach of chemical reduction of silver ions in PVP and surfactants. Nonetheless, gamma irradiation is one of the most promising methods to produce Ag NPs at room temperature, using fewer reducing agents and byproducts. Increasing the γ-irradiation doses from 1 to 5 kGy enhanced the concentration of Ag NPs, indicated by UV–vis spectroscopy [9].

Gamma irradiation is still gaining a lot of popularity in medicine [10], the clothing industry [11], grafting [12], and even heritage artefact conservation [13]. In the work of Geba et al. [14] it was concluded that gamma irradiation at 10 kGy of accelerated aged silk and wool fabrics did not cause significant changes of their physical and chemical features.

Nanoparticles have a worldwide use, with an increasing trend in production every year [15]. These materials are used in medicine, from devices [16] to drug delivery [17], electronics [18], and textiles [19]. Because of their heavy usage around the world, research is being conducted to reduce production cost, modify physical or chemical properties, or create new material types. Antibacterial fabrics were obtained by Truong et al. [20] by gamma irradiation of cotton materials in an AgNO_3_ chitosan solution.

The study’s aim was to propose replacing the high-energy consumption, classical textile finishing methods, Ref. [21] used to fix the enhancing substance on the textile with the more ecological gamma technologies. For this objective, we chose to develop a basic set of methods for creating antibacterial textiles with gamma irradiation and nanoparticles combined treatment. The novelty of the study consists of preparing new antibacterial cotton textile materials by various nanoparticles and gamma irradiation treatments.

## 2. Materials and Methods

### 2.1. Silver and ZnO Nanoparticles Preparation

#### 2.1.1. Ag Nanoparticles Were Prepared by Two Different Methods, Using Chitosan and PVP, Respectively

The Ag/chitosan (Ag/C) nanoparticles were prepared with a chitosan aqueous solution (concentration 0.2%), which was prepared by dissolving the polymer powder in 1% acetic acid under magnetic stirring (300 rpm) at room temperature for 12 h. The chitosan used was a product with average viscosity, molecular mass = 300 kDa, and an 82% degree of deacetylation (Sigma-Aldrich, St. Louis, MO, USA). In the chitosan solution, different volumes of AgNO_3_ (0.1 M) were added as was 0.1 mL of NaOH 1 M for adjusting the pH and speeding up the reduction reaction. The mixture was left to react for 8 h at 90 °C. The verification of the nanoparticle obtaining was done by UV-Vis spectrophotometry; data are available in the Supplementary Material.

Ag/PVP nanoparticles were obtained by the hydrothermal method by reducing silver salts. An AgNO_3_ solution with a 0.1 M concentration was prepared, and 1 mL of NaBH4 0.5 M was added under strong stirring (300 rpm) for the reduction of Ag^+^ ions. Afterwards, the Ag nanoparticles were dispersed in a 2% polyvinylpyrrolidone solution. The schematic chemical structure of the composites is given in the Supplementary Materials along with their characterization.

#### 2.1.2. Zinc Oxide Nanoparticles Preparation

The ZnO nanoparticles (ZnO/PVP) were obtained by hydrothermal synthesis at a normal pressure and moderate temperature. A total of 100 mL of Zn(NO_3_)_2_·6H_2_O with a 0.5 M concentration was introduced into a 3 pits balloon, and 50 mL of NaOH was added under vigorous stirring (300 rpm) at 65 °C. The mixture became opalescent immediately, and stirring was continued for 8 h. Subsequently, the resulting suspension was filtered and washed 2–3 times with distilled water and alcohol. To increase the crystallinity factor, the ZnO nanopowder was calcined in the oven at 150 °C for 4 h. Then, the ZnO nanoparticles were dispersed in a polyvinylpyrrolidone 2% solution. Characterization of the nanoparticles is given in the Supplementary Materials.

### 2.2. Textile Materials Treatment

Bleached, plain weave 100% cotton fabric with a weight per unit area of 168 g/m^2^ was used for the functionalization process. Sixteen samples were cut; one was kept as a reference (R), and 15 samples, i.e., 5 for each type of prepared composites, were impregnated using ultrasonic bath. One of the 5 impregnated samples was kept as an impregnated control (M), 2 were irradiated while still wet (W) at 5 kGy and 20 kGy (5W and 20W), respectively, and another 2 were irradiated at 5 kGy and 20 kGy in a dry state after drying at room temperature (5D and 20D).

#### Gamma Irradiation

The irradiation treatments were conducted at the IRASM Centre of Technological Irradiations of the Horia Hulubei National Institute for Physics and Nuclear Engineering. The cotton textiles were irradiated in a Gamma Cell 5000 research unit that uses Co-60 sources at a dose rate of 3.3 kGy/h. The doses were measured using the ECB (ethanol-chlorobenzene) method using ISO/ASTM 51538:2009. The uncertainty of the absorbed dose measurement was 5%, and the dose uniformity ratio (ratio between delivered maximum and minimum absorbed dose) was 1.1.

### 2.3. Materials Characterization

The cotton samples were characterized before and after irradiation and nanoparticles treatment by FTIR spectroscopy and thermal analysis. A Netzsch STA 409 PC Luxx Simultaneous Thermal Analyzer with a TG/DSC sample carrier was used for testing under an inert atmosphere (50 mL/min pure nitrogen). The tests were performed from 40 °C to 600 °C at 10 K/min in aluminum crucibles with pierced caps. The sample mass was about 10 mg. Each measurement was performed in triplicate. For thermal analysis, the cotton samples were cut in discs of about 5.5 mm (close to the size of the Al crucibles).

The FTIR spectra were obtained with a Vertex 70 equipment (Bruker Optics) as an average of 5 measuring points in the ATR mode using a diamond crystal accessory. The spectra were acquired with OPUS software for the range of 400–4000 cm^−1^ with 64 scans and were adjusted by vector normalization and baseline correction, available in the OPUS software package.

The antimicrobial activity of the treated textiles was tested against *Pseudomonas aeruginosa* (ATCC 9027) and *Staphylococcus aureus* (ATCC 6538) by the disc diffusion test. The textile samples were placed on Mueller–Hinton sterilized agar, and the agar plate was inoculated uniformly with 108 UFC/mL; lastly, the plates were incubated at 36 °C for 24 h. The bacteria developed and enveloped the medium, except the resulting sterile area named “inhibition zone”, generated by the antimicrobial textiles. After 24 h, the inhibition zone diameter was measured.

## 3. Results and Discussion

### 3.1. FTIR-ATR Analysis

**Figure 1:**
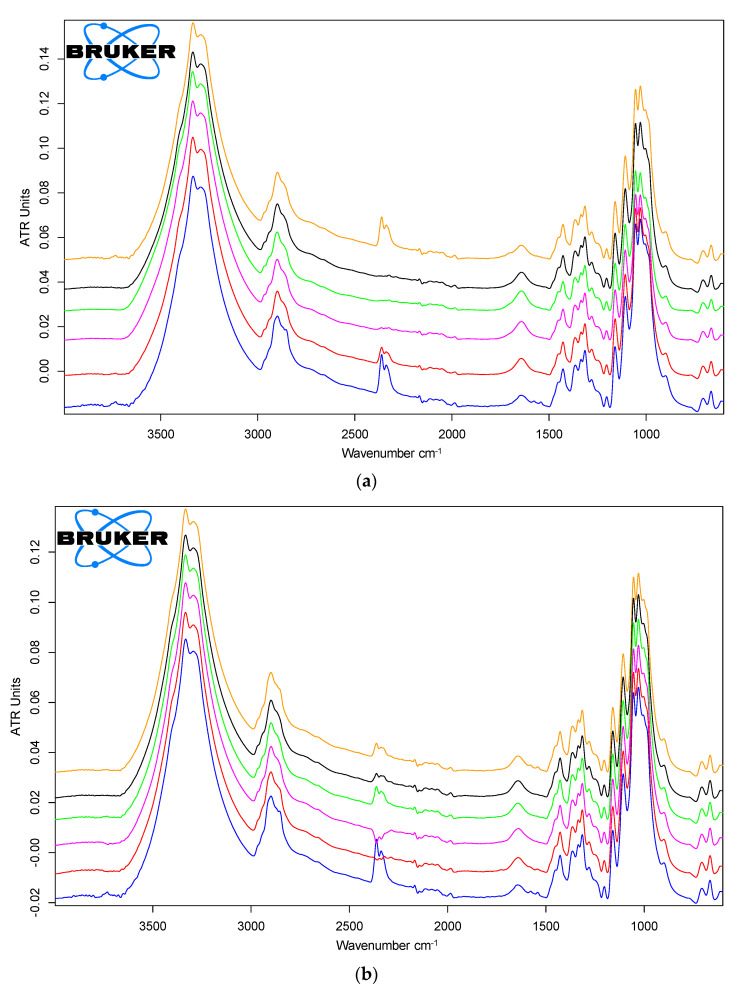

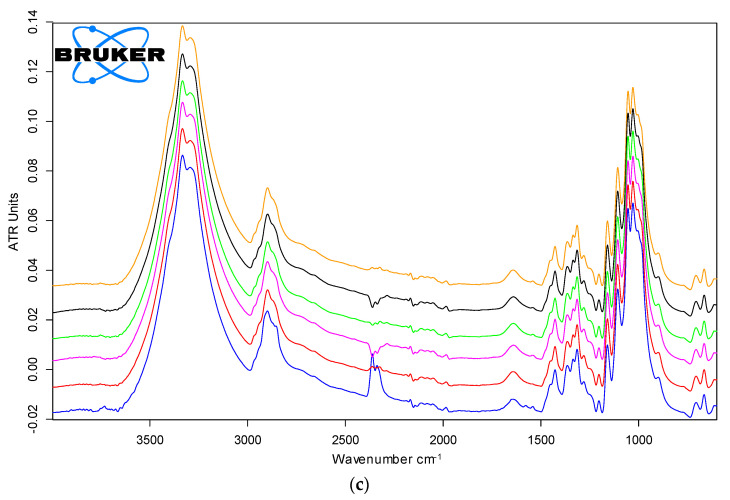
FTIR-ATR spectra: (**a**) unimpregnated and unirradiated cotton material reference (blue line); impregnated with Ag/C and unirradiated (red line—control 0 kGy); and impregnated and irradiated at 5 kGy in a dry state (purple line) and a wet state (green line) and 20 kGy in a dry state (black line) and a wet state (orange line); (**b**) unimpregnated and unirradiated cotton material reference (blue line); impregnated with Ag/PVP and unirradiated (control 0 kGy—red line); and impregnated and irradiated at 5 kGy in a dry state (purple line) and a wet state (green line) and 20 kGy in a dry state (black line) and a wet state (orange line); (**c**) unimpregnated and unirradiated cotton material reference (blue line); impregnated with ZnO/PVP and unirradiated control 0 kGy (red line); and irradiated at 5 kGy in a dry state (purple line) and a wet state (green line) and 20 kGy in a dry state (black line) and a wet state (orange line).

FTIR spectra of reference cotton, impregnated, and gamma-irradiated textiles are shown in Figure 1a–c.

No major modification in the cellulose textile FTIR spectra was induced by the presence of the nanocomposites or gamma irradiation as may be seen from Figure 1a–c. In Table 1, the main peaks of cotton cellulose and some of its impurities are presented based on the references from Gilbert et al. [22] and Chung et al. [23].

The broad peak of 3550–3100 cm^−1^ is attributed to the OH stretch, and the one at 2980–2800 cm^−1^ is the CH stretch. A first observation is the presence of the 2850 cm^−1^ peak correlated to the symmetric CH2 stretch and of a small shoulder at around 2916 cm^−1^ corresponding to the asymmetric CH2 stretch, related to the presence of waxes in the cotton. There are also two other peaks that appear at 1577 cm^−1^ and 1542 cm^−1^ that are attributed to impurities, and due to amide II, aromatic C=C stretching vibrations, or C-O vibrations from the COOR group. Due to their small intensity, all the above peaks of impurities were no more visible in the FTIR spectra of cotton materials after the nanocomposites’ application and irradiation.

Because of the low concentration of chitosan and PVP and the fact that many of their peaks overlap with the cellulose peaks, their presence could not be detected directly but indirectly as a decrease in the intensity of cellulose peaks. Differences in the case of peak intensities are small, around ±0.005 ATR units for the Ag/PVP and ZnO/PVP samples in all their variants, but for the Ag/C samples, there is a marked reduction in intensity for the 1054, 1030, and 1001 cm^−1^ peaks. All newly obtained materials have a similar tendency of a higher 3550–3100 peak intensity that may have been caused by increased hydrophilicity and a lower intensity for the 1054, 1030, and 1001 cm^−1^ peaks. Peak shifts are around ±0.5 cm^−1^, which shows that there are no major changes in the chemical structure for the cotton textile caused by the applied treatments.

The main spectral differences can be observed for the analyzed textile materials, especially for the C-O-C vibration mode of cellulose at about 1004 cm^−1^. The most important spectral changes, i.e., decrease of cellulose band intensity, is observed amid the control and the material impregnated and irradiated with Ag/chitosan nanoparticles, showing the adhesion of the nanoparticles on the material surface.

### 3.2. TG/DTG Analysis

**Figure 2:**
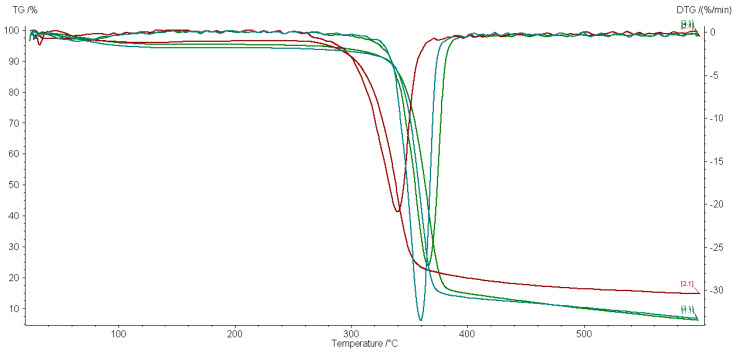
TG/DTG data of cotton impregnated with Ag/C (green lines), Ag/PVP (red lines), and ZnO/PVP (blue lines) and irradiated at 5 kGy in a dry state.

**Figure 3:**
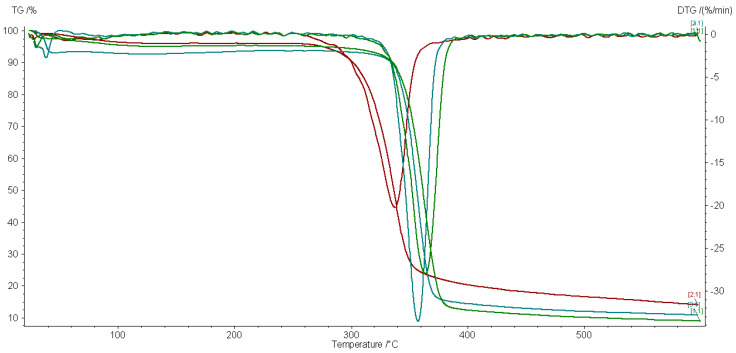
TG/DTG analysis of cotton impregnated with Ag/C (green lines), Ag/PVP (red lines), and ZnO/PVP (blue lines) and irradiated at 5 kGy in a wet state.

Selected TG/DTG curves of impregnated and 5 kGy gamma-irradiated textiles are shown in Figure 2 and Figure 3.

The TG/DTG tests were done to observe the effect of our ecological gamma alternative treatment on the properties of the material. Each nanocomposite affects the thermal behavior of the cotton textile to a different extent. Table 2 displays that the irradiation does not produce the same effect on each material in a dry or wet state. Ag/C-treated samples have the lowest residual mass and Ag/PVP the biggest residual mass in correlation with a lower thermal stability as revealed by the decomposition temperature.

The TG/DTG curves shown in Figure 2 and Figure 3 have only one decomposition peak corresponding to the decomposition of cellulose materials [24]. The control sample has a maximum decomposition temperature at 365.2 °C, which is close to the decomposition temperature from the literature [24,25].

Both dry- and wet-state-treated samples have similar TG and DTG curves but with small differences: the wet-state-irradiated samples have a lower maximum decomposition temperature of around 2–3 °C while almost all the dry-state-treated samples have a lower thermal stability compared to the control group. The Ag/C-treated material is the one with the closest thermal parameters to the original materials. In a wet state, the radiolysis processes produce more reactive oxygen than in a dry state. These short life reactive species may further interact with the cotton support material. The thermal parameters of samples irradiated at 20 kGy have an even smaller variation toward the reference sample, as expected, due to the predominance of crosslinking effects against bond scission, which prevails at lower doses.

### 3.3. Antimicrobial Testing

**Figure 4:**
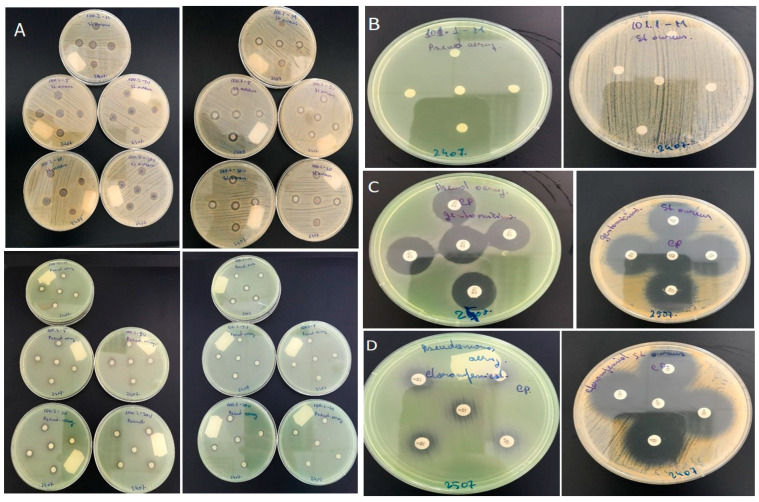
The antimicrobial effect of samples Ag/C, Ag/PVP, and ZnO/PVP: (**A**) antimicrobial effect of samples Ag/C and Ag/PVP on bacterial strains of *Staphylococcus aureus* (upper photos) and *Pseudomonas aeruginosa* (lower photos); (**B**) antimicrobial action of sample ZnO/PVP (M) against *Pseudomonas aeruginosa* (left) and *Staphylococcus aureus* (right); (**C**) antimicrobial action of gentamicin 10 mg against the two microorganisms tested, *Pseudomonas aeruginosa* (left) and *Staphylococcus aureus* (right); (**D**) antimicrobial action of chloramphenicol 30 mg against the two microorganisms tested, *Pseudomonas aeruginosa* (left) and *Staphylococcus aureus* (right).

The antimicrobial activity of tested samples using the disk diffusion method are represented in the Figure 4, where nanoparticles impregnated textile disks were inoculated on Muller–Hinton agar plates with a *Staphylococcus aureus* and *Pseudomonas aeruginosa* 24 h fresh suspension.

**Figure 5:**
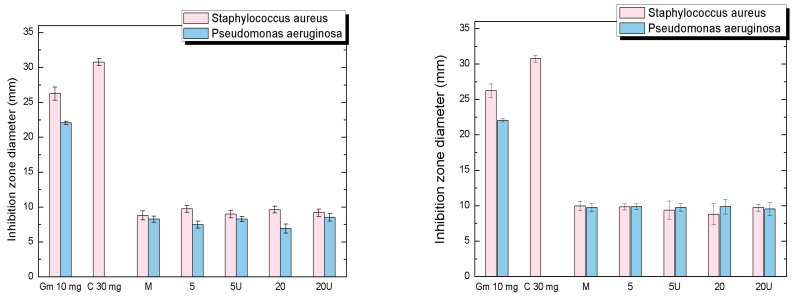
Antimicrobial effect of Ag/C (**left**) and Ag/PVP (**right**) textile samples (control–M and irradiated at 5 and 20 kGy in a dry and wet state (u)) compared to gentamicin 10 mg (Gm 10 mg) and chloramphenicol 30 mg (C 30 mg).

Two potent antibiotics, gentamicin 10 mg and chloramphenicol 30 mg, were used as positive controls to compare the antimicrobial activity of textile samples. While chloramphenicol 30 mg managed to inhibit only the growth of *Staphylococcus aureus*, gentamicin 10 mg showed a good inhibition zone for both tested microorganisms (see Figure 5).

Both Ag/C and Ag/PVP, in all tested forms (M, 5D, 5W, 20D, 20W), exhibit an antimicrobial effect against *Staphylococcus aureus* and *Pseudomonas aeruginosa*, comparable with the control.

All variants of Ag/C display a strong effect on *Staphylococcus aureus*, with the inhibitory zones ranging from 9–10 mm. *Pseudomonas aeruginosa* displays slightly less sensitivity to Ag/C nanoparticles, with inhibitory areas of 7–8 mm.

The sample Ag/PVP, in all tested forms, displays similar antimicrobial inhibitory capacity for both tested microorganisms, with inhibition zones around 9–10 mm.

Both samples did not exhibit significant antimicrobial inhibitory differences between tested variances. Similar results were obtained by Truong et al. [20].

The fact that the samples (textiles) impregnated with ZnO/PVP nanoparticles had no antimicrobial activity on tested microorganisms is surprising. The experiments will be continued using higher nanoparticles concentrations and other microorganisms.

## 4. Conclusions

While the nanocomposites’ presence has been identified indirectly by FTIR-ATR spectroscopy, thermal analysis proved the presence of the nanoparticles on the material along with the antimicrobial testing that emphasized that there is an important antimicrobial effect produced by Ag/C and Ag/PVP nanoparticles for the two selected studied microorganisms.

Compared to the standardized antibiotics disk used as a positive control, our materials show promising results, their antimicrobial capacity being around 25–30% of the tested standardized antibiotics (gentamicin 10 mg and chloramphenicol 10 mg). Further investigations should be performed on various concentrations and other pathogens, such as *E. coli*, *Salmonella* sp., and *Klebsiella*.

Small differences in the IR peak intensities prove that the selected doses of gamma irradiation do not have a major impact on the physical-chemical properties of the cotton textile material. The small decrease in thermal stability of the samples at 5 kGy gamma irradiation dose is observed in correlation with the known predominance effects of gamma irradiation of bond breaking at low doses and crosslinking at higher doses.

From a preliminary perspective, the experiment proved to be a success as we obtained antibacterial textiles while also providing some additional information about the interaction of gamma irradiation with the nanoparticles impregnated cotton material.

These findings support a starting point for further research in utilizing gamma technologies as a more ecological method to the classical textile finishing methods and possibly even as an alternative for the other textile processing steps.

## Figures and Tables

**Table 1:** FTIR data of cotton cellulose.

Experimental FTIR-ATR (cm^−1^)	Literature (cm^−1^)	Vibration Mode
3550–3100	3570–3200	H-bonded OH stretch
2980–2800	3000–2800	CH stretch
2850	2849	Symmetric CH_2_ stretch; long alkyl chain
1642	1650–1633	H_2_O adsorbed
1428	1430	CH wagging in plane bending
1368	1372	CH bending deformation stretch
1335	1336	OH in plane bending
1314	1320	CH wagging
1280	1282	CH deformation stretch
1247	1236	OH in plane bending
1204	1204	OH in plane bending
1161	1178	Asymmetric bridge COC
1108	1130	Asymmetric bridge COC
1054	1092	Asymmetric in plane ring stretch
1030	1042	CO stretch
1001	998–1002	CO stretch
898	898	Asym. out of phase ring stretch C_1_OC_4_; β glycosidic bond

**Table 2:** Comparative TGA data, treated by NP impregnation and irradiation cotton samples.

Treatment	5 kGy Irradiation in a Dry State		5 kGy Irradiation in a Wet State	
	Decomposition Peak	Residual Mass	Decomposition Peak	Residual Mass
Ag/C	366.4 °C 27.18%/min	6.13%	364.0 °C 27.97%/min	8.86%
Ag/PVP	340.0 °C 20.93%/min	14.68%	337.6 °C 20.28%/min	14%
ZnO/PVP	360.0 °C 33.50%/min	6.71%	358.1 °C 32.30%/min	11.95%

## Data Availability

Data are available on request from the authors.

## Supplementary Materials

The following supporting information can be downloaded at: https://www.mdpi.com/article/10.3390/ma15082734/s1, Figure S1: Chemical structure of the Ag/chitosan, Ag/PVP and ZnO/PVP composites, Figure S2: UV-Vis spectrum of dispersion of Ag NPs in 0.2% Chitosan polymer solution(right) the image of dispersion of Ag NPs prepared in Chitosan solution 0.2% after 48 h (left), Figure S3: SEM image of the ZnO nanopowder prepared by the hydrothermal method (a) and the size and size distribution of the nanoparticles for the sample obtained at 65 °C, before calcination.
