# Supplementary material for: Gamma Irradiation and Ag and ZnO Nanoparticles Combined Treatment of Cotton Textile Materials
Source: Materials (Basel). 2022 Apr 8;15(8):2734. doi: 10.3390/ma15082734 (PMC9031458; doi:10.3390/ma15082734)
Supplement: Supplementary file 1 [file materials-15-02734-s001.zip › materials-1557878-supplementary.pdf]

Supplementary data

Gamma Irradiation and Ag and ZnO Nanoparticles Combined Treatment of Cotton Textile Materials

Ovidiu-Alexandru Capraru, Bogdan Lungu, Marian Virgolici, Mihai Constantin, Mihalis Cutrubinis, Laura Chirila, Ludmila Otilia Cinteza and Ioana Stanculescu

The schematic chemical structure of the obtained Ag/chitosan, respectively Ag/PVP and ZnO/PVP nanoparticles (NP) is given in the Figure S1.

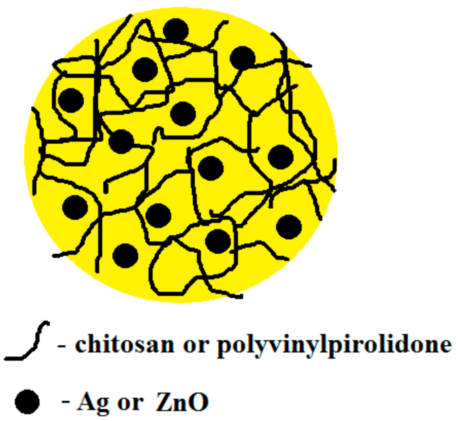

Figure S1. Chemical structure of the Ag/chitosan, Ag/PVP and ZnO/PVP composites.

To confirm the formation of nanoparticles, the absorption spectrum of Ag NPs dispersions was measured using a UV-Visible spectrophotometer Shimadzu UVmini 240. Figure S2 shows the UV-Vis spectrum of a dispersion of Ag NPs prepared in 0.2% chitosan solution at a final concentration of 0.03 M $AgNO_3$ precursor in the reaction medium.

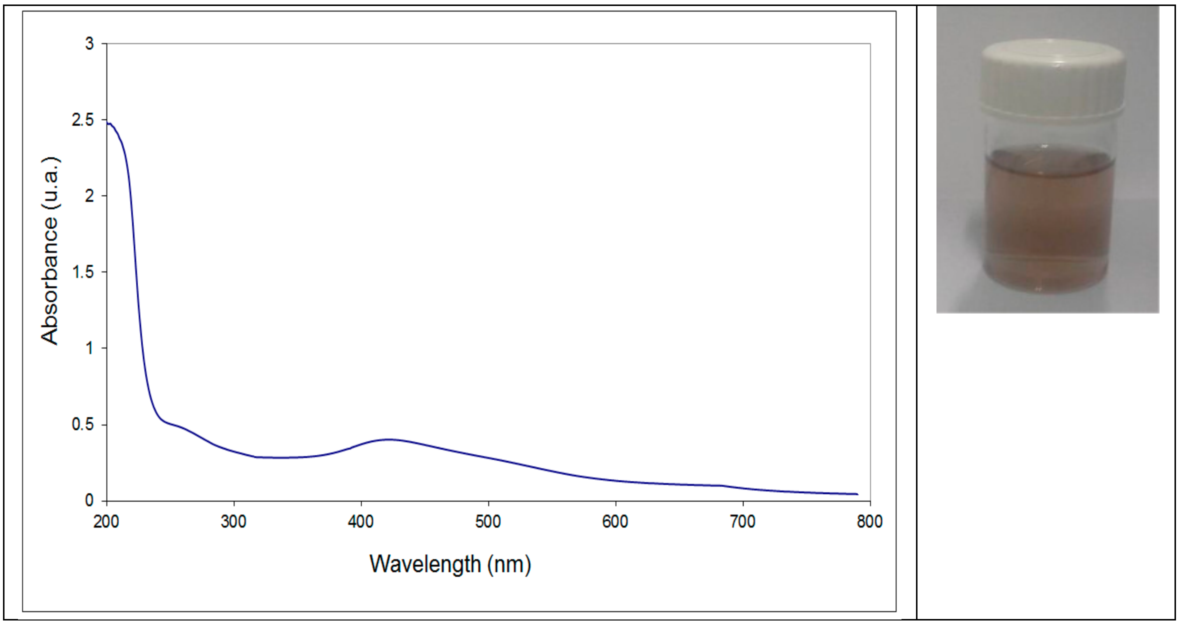

Figure S2. UV-Vis spectrum of Ag NPs dispersion in 0.2% chitosan polymer solution (right); image of of Ag NPs dispersion prepared in chitosan solution 0.2% after 48 h (left).

The reaction temperature plays an important role and strongly influences the particle size and the dispersion of AgNP in the chitosan solution. The reduction reaction, respectively the growth of Ag particles, takes place much more slowly at low temperatures and in the presence of the biopolymer as a reducing agent, due to the much weaker effect of its functional groups. This solution was chosen because the procedure of gamma radiation irradiation of textiles materials, performed for various purposes, such as bacterial decontamination, sterilization, functionalization, etc. can create the conditions for the much faster development of the metal precursor reduction reaction, and respectively the much faster "in situ" formation of Ag NPs directly on the surface of the treated materials.

Synthesis and characterization of the ZnO nanoparticles in polyvinyl pyrrolidone

Polyvinyl pyrrolidone (PVP) was chosen as the matrix for the formation of the composite material, a polymer which, in addition to its low toxicity, also has the advantage of wetting and filmogenicity properties, which make it a widely used ingredient in coating formulations.

The ZnO nanoparticles obtained have a relatively spherical shape, according to SEM images and average dimensions in the range of 280-520 nm as measured by the DLS method (Fig. S3). The size of the obtained NPs was measured by the Dynamic Light Diffusion (DLS) method on a Zetasizer Nano, Malvern instrument.

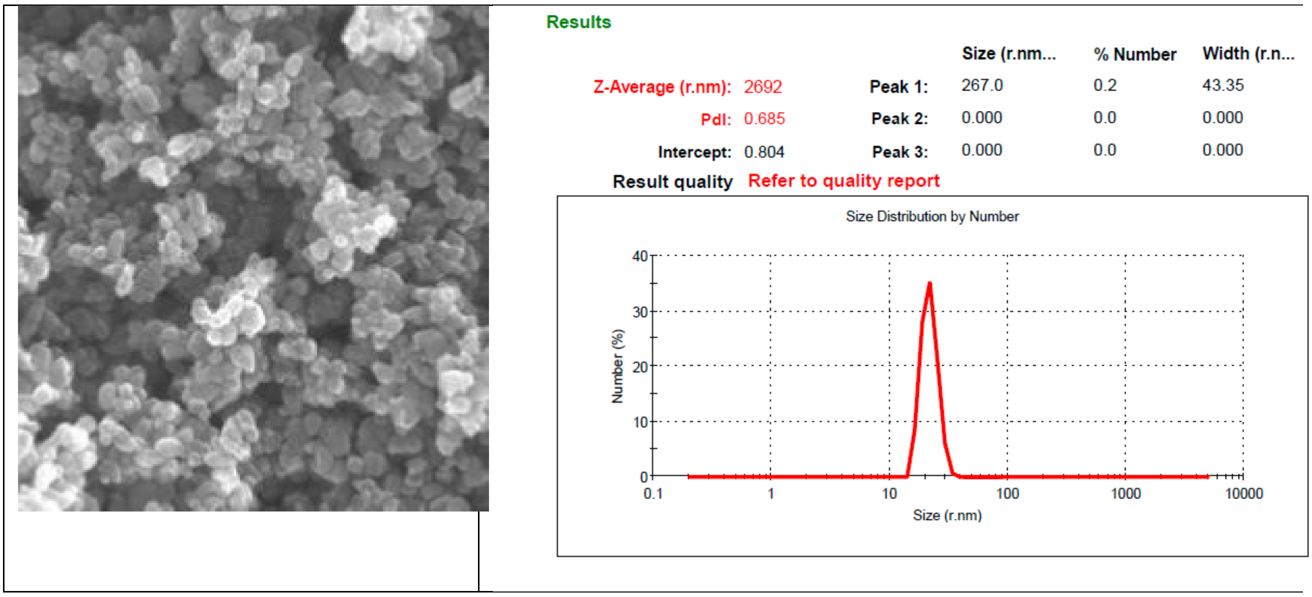

Figure S3. SEM image of the ZnO nanopowder prepared by the hydrothermal method (right); the size and size distribution of the nanoparticles for the sample obtained at 65 ° C, before calcination (left).
